# Generation of Tandem Direct Duplications by Reversed-Ends Transposition of Maize *Ac* Elements

**DOI:** 10.1371/journal.pgen.1003691

**Published:** 2013-08-15

**Authors:** Jianbo Zhang, Tao Zuo, Thomas Peterson

**Affiliations:** Department of Genetics, Development and Cell Biology, Department of Agronomy, Iowa State University, Ames, Iowa, United States of America; University of Utah School of Medicine, United States of America

## Abstract

Tandem direct duplications are a common feature of the genomes of eukaryotes ranging from yeast to human, where they comprise a significant fraction of copy number variations. The prevailing model for the formation of tandem direct duplications is non-allelic homologous recombination (NAHR). Here we report the isolation of a series of duplications and reciprocal deletions isolated *de novo* from a maize allele containing two Class II *Ac/Ds* transposons. The duplication/deletion structures suggest that they were generated by alternative transposition reactions involving the termini of two nearby transposable elements. The deletion/duplication breakpoint junctions contain 8 bp target site duplications characteristic of *Ac/Ds* transposition events, confirming their formation directly by an alternative transposition mechanism. Tandem direct duplications and reciprocal deletions were generated at a relatively high frequency (∼0.5 to 1%) in the materials examined here in which transposons are positioned nearby each other in appropriate orientation; frequencies would likely be much lower in other genotypes. To test whether this mechanism may have contributed to maize genome evolution, we analyzed sequences flanking *Ac/Ds* and other *hAT* family transposons and identified three small tandem direct duplications with the structural features predicted by the alternative transposition mechanism. Together these results show that some class II transposons are capable of directly inducing tandem sequence duplications, and that this activity has contributed to the evolution of the maize genome.

## Author Summary

The recent explosion of genome sequence data has greatly increased the need to understand the forces that shape eukaryotic genomes. A common feature of higher plant genomes is the presence of large numbers of duplications, often occurring as tandem repeats of thousands of base pairs. Despite the importance of gene duplications in evolution and disease, the precise mechanism(s) that generate tandem duplications are still unclear. In this study we identified nine new spontaneous duplications that arose flanking elements of the *Ac* transposon system. These duplications range in size from 8 kbp to >5,000 kbp, and all cases exhibit features characteristic of *Ac* transposition. Using similar criteria in a bioinformatics search, we identified three smaller duplications adjacent to other *hAT* family transposons in the maize B73 reference genome sequence. Our results show that transposable elements can directly generate tandem duplications via alternative transposition, and that this mechanism is responsible for at least some of the duplications present in the maize B73 genome. This work extends the significance of Barbara McClintock's discovery of transposable elements by demonstrating how they can act as agents of genome expansion.

## Introduction

In addition to generating additional copies of coding sequences that can be used as substrates for gene evolution [1], gene duplication may also cause immediate phenotypic impacts such as human disease [2]. Segmental duplications (SD)–two or more chromosomal segments with high homology–are common in higher plant and animal genomes. In humans and mice, ∼5% of the genome is composed of segmental duplications (≥90% in identity and ≥1 kb in length); tandem duplications (direct and inverted) account for 35.2% and 21.6% of the total duplications in the mice and human genomes, respectively [3], [4]. Many plants contain an even higher percentage of duplicated sequences. In rice, segmental duplications comprise 15–62% of the genome, depending on the sequences compared and classification criteria employed [5]–[8]. Moreover, ca. 29% of rice genes are arranged in tandem repeats [9]. Recent studies by others have also confirmed the presence of numerous duplicated sequences in the maize genome [10]–[14]. Comparison of genome sequences from different individuals of the same species revealed that copy number variation (CNV) is widespread, and that tandem duplications account for a significant proportion of the observed CNV. In Arabidopsis and maize, more than 50% of CNV segments contain tandem duplications [15]–[17]. In cattle and mice, copy number “gain” CNVs are predominantly associated with tandem local duplications, rather than interspersed duplications [18]. These observations indicate that CNVs and associated tandem duplications are contributing to rapid genome evolution.

There are several mechanisms proposed to generate tandem duplications, including 1) non-allelic homologous recombination (NAHR) between short repeats flanking a DNA segment [19], [20]; 2) break-induced replication (BIR) [21], [22] which can be mediated by short microhomology regions [19], [23]; and 3) fork stalling and template switching (FoSTeS) [24]. Here, we investigated the potential role of Class II transposable elements in directly generating tandem sequence duplications via aberrant transposition reactions.

The standard model for transposition of DNA elements involves excision of the termini of a single transposon from a donor locus and reinsertion into a target site; the net effect is the movement of the element, without any other changes to the genome. In contrast, Alternative Transposition (AT) events involve the termini of two separate, usually nearby elements. AT reactions can generate a variety of genome rearrangements; for example, the Drosophila *P* element system can undergo Hybrid Element Insertion (HEI) events that produce a wide array of flanking rearrangements [25]–[27] In maize, the *Ac/Ds* transposable element system is known to undergo at least two types of AT events that lead to genome rearrangements. First, Sister Chromatid Transposition (SCT) involves the directly-oriented 5′ and 3′ termini of closely-linked elements located on sister chromatids. Depending on the location of the transposition target site, SCT can generate chromatid bridges and breaks [28], [29], as well as flanking inverted duplications and deletions [30]. Second, Reversed Ends Transposition (RET) involves the reversely-oriented 5′ and 3′ termini of two elements located nearby each other on the same chromatid. In addition to bridges and breaks [29], RET can generate flanking inversions, deletions, permutations, and reciprocal translocations [31], [32]. An additional type of AT event termed Single Chromatid Transposition (SLCT) which involves the directly-oriented 5′ and 3′ termini of nearby elements on the same chromatid has been observed in transgenic rice containing maize *Ac/Ds* elements, but this reaction was not detected in maize [33].

We predicted that RET may also generate tandem direct duplications. Here we show that a single pair of reversed *Ac* termini induced a series of nine flanking tandem duplications ranging in size from 8157 bp to ∼5.3 Mbp. The structures of these tandem duplications and their associated deletions strongly indicate that they were indeed generated by reversed *Ac* ends transposition. Moreover, we identified three tandem duplications flanking other *hAT* transposons with the features predicted by RET in the maize B73 reference genome sequence.

## Results

### Identification of duplication candidates from maize twin sectors

**Figure 1:**
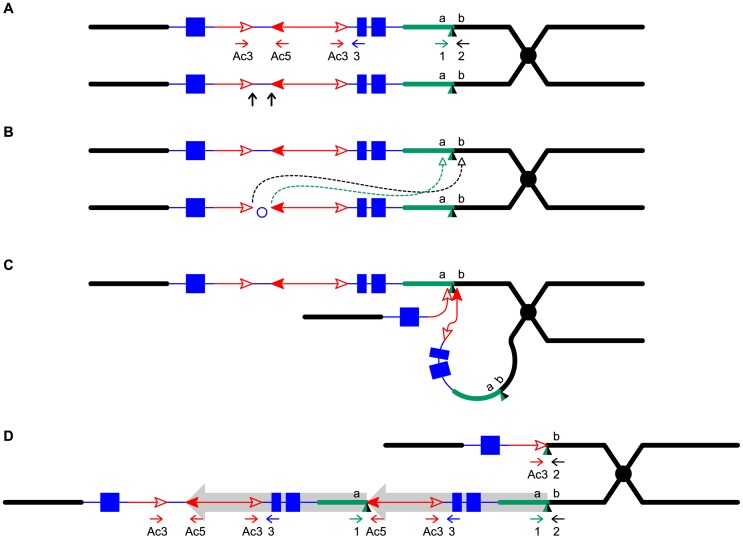
Reversed *Ac* ends transposition generates direct duplication. The two lines indicate sister chromatids of maize chromosome 1, joined at the centromere (black). The blue boxes are exons 3, 2, and 1 (left to right) of the *p1* gene. Red lines with arrowhead(s) indicate *Ac/fAc* insertions, and the open and solid arrowheads indicate the 3′ and 5′ ends, respectively, of *Ac*/*fAc*. The short horizontal arrows show the orientations and approximate positions of PCR primers, and the numbers below are the primer names. The green/black triangles indicate the transposon target site sequences and target site duplications. (*A*) *Ac* transposase cleaves the lower chromatid at the 3′ end of *fAc* and the 5′ end of *Ac* (arrows). (*B*) Following transposase cleavage, the internal *p1* genomic sequences are joined to form a circle. Dotted lines indicate the insertion of the *fAc* and *Ac* termini into the a/b site on the sister chromatid. (*C*) Transposon ends insert into the upper sister chromatid at a proximal site. (*D*) The *Ac* 5′ end joins to the distal side (green) of the target site and the *fAc* 3′ end joins to the proximal side (black) of the target site to generate a proximal deletion (upper chromatid) and a direct duplication (lower chromatid). The shaded arrows encompass the duplicated segments. For animation, please see Movie S1.

**Figure 2:**
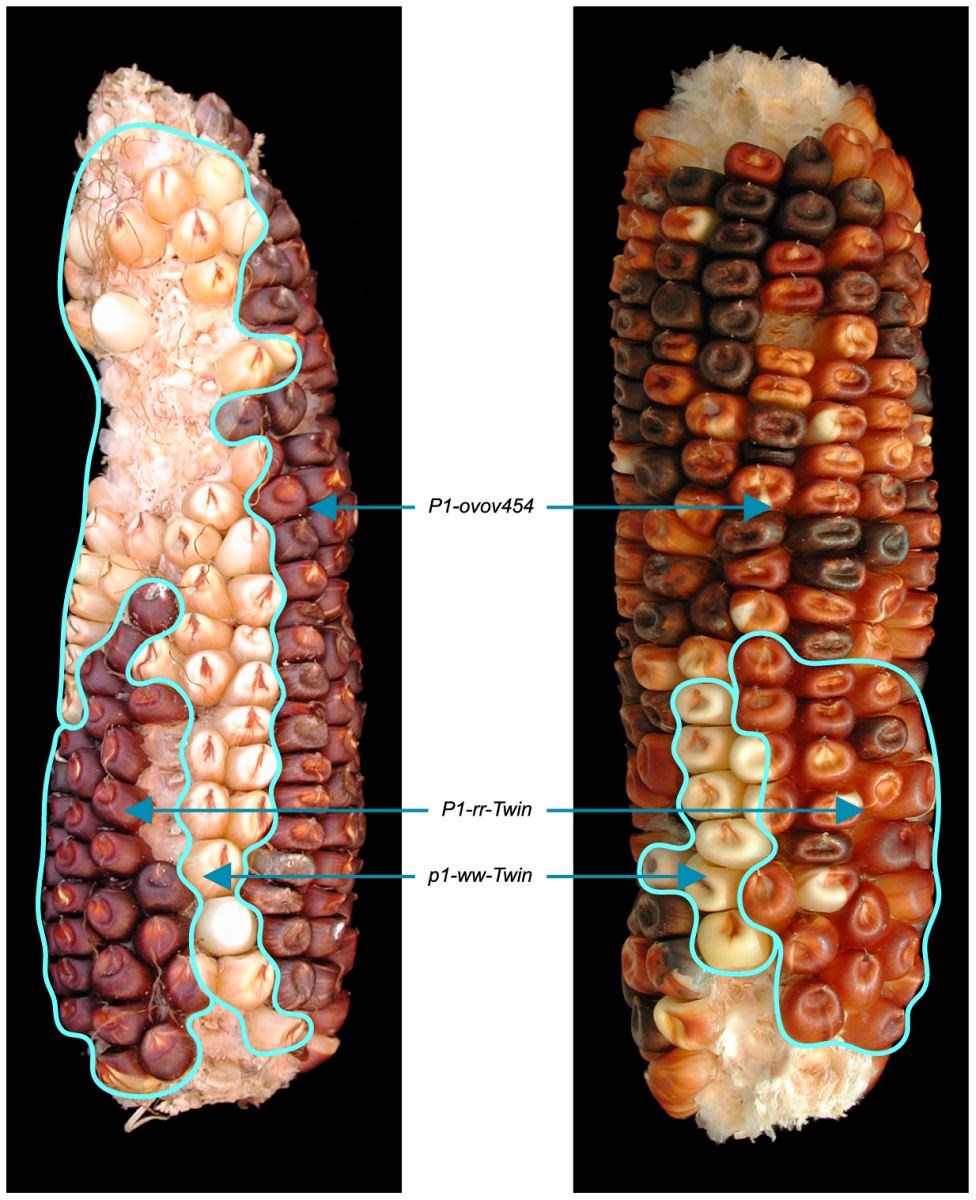
Ears with twinned sectors T1 (left) and T481 (right). The white and red phenotypic twinned sectors are outlined. The remainder of the ear has the orange-variegated phenotype specified by the progenitor *P1-ovov454* allele.

To detect newly-formed duplications, we screened maize materials that contain elements of the *Ac/Ds* transposon system inserted into the *p1* gene that controls kernel pericarp (seed coat) pigmentation. We initiated the screen with the progenitor allele *P1-ovov454*, which carries a pair of reversely-oriented *Ac* termini in the *p1* gene intron 2 (Figure 1*A*). If transposition of the reversed *Ac* ends occurs during DNA replication and the excised termini insert into the sister chromatid, two unequal chromatids can be generated: one chromatid contains a tandem direct duplication, and the other contains a corresponding deletion (Figure 1*D*, lower and upper chromatids, respectively; for animated version please see Movie S1). These two chromatids will segregate into two adjacent daughter cells at mitosis; further mitotic divisions could generate a visible twinned sector. The new mutant chromosomes can be transmitted through meiosis to the kernels within the sectors and subsequently propagated as heritable alleles. Because the *P1-ovov454* allele specifies orange variegated pericarp and orange variegated cob, both gains and losses of *p1* expression can be recognized. The sector containing the deletion chromosome (white twin, *p1-ww-Twin*) would have white (colorless) pericarp due to loss of *p1* gene exons 1 and 2, while the sector with the duplication chromosome (red twin, *P1-rr-Twin*) would contain two copies of *Ac* and exhibit fewer red and white stripes due to the negative *Ac* dosage effect [34], [35] (see Methods for details). We screened ∼2000 *P1-ovov454*/*p1-ww* ears and identified six ears with this type of twinned sector. Two such ears which gave rise to duplication alleles *P1-rr-T1* and *P1-rr-T481* are shown in Figure 2; the remaining four twin sector ears gave rise to more complex rearrangements which are still under investigation.

### The red twin carries a tandem direct duplication

**Figure 3:**
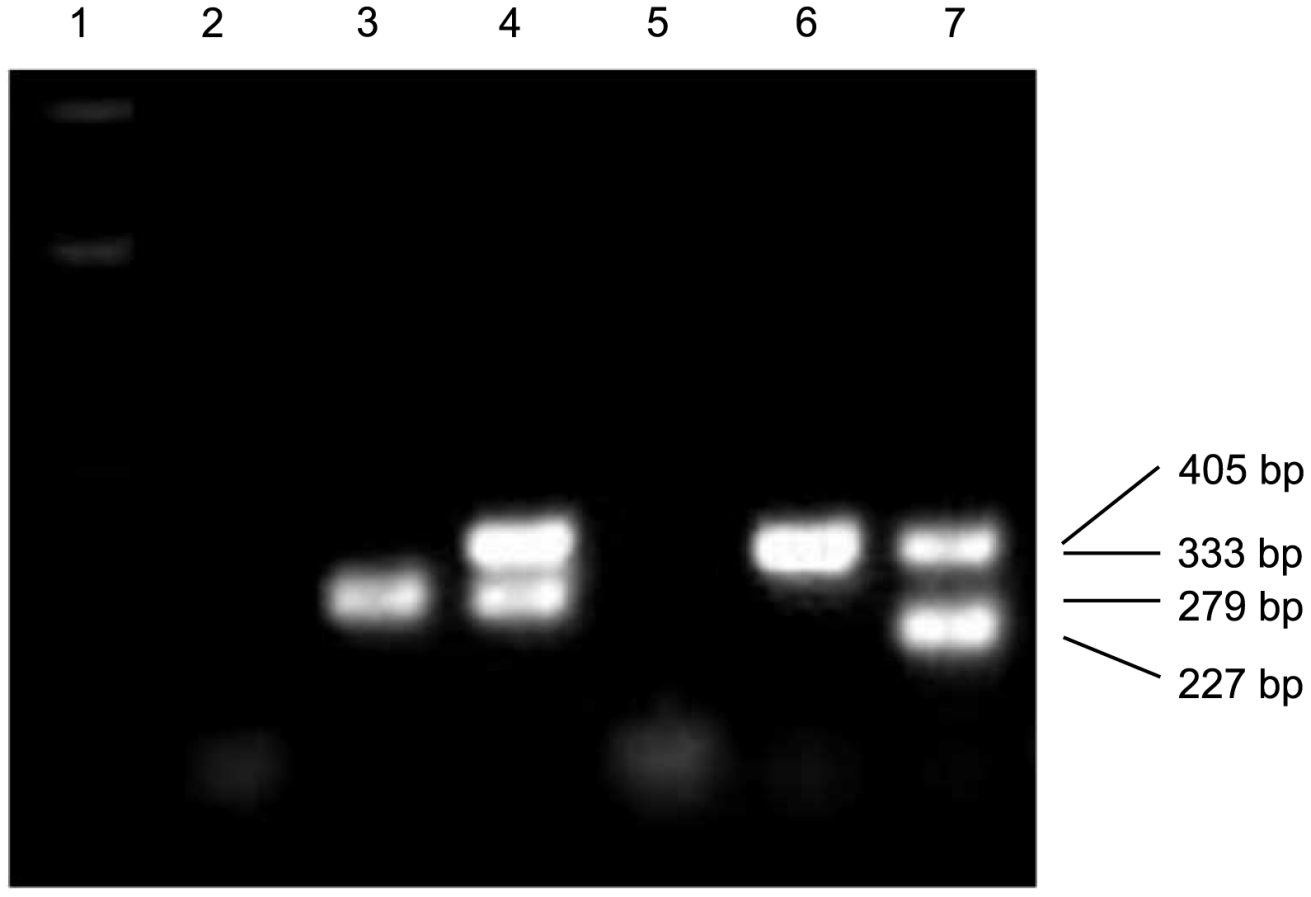
PCR analysis of the twinned alleles with primers 1+2+Ac5. Lane 1: DNA ladder; lanes 2 and 5: water (negative control); lanes 3 and 4: *P1-ovov454*, *P1-rr-T1*; lanes 6 and 7: *P1-ovov454*, *P1-rr-T481*. Note that primers 1 and 2 are specific for each allele.

The RET model (Figure 1) predicts that the breakpoints of the duplication alleles (sequence *a* in Figure 1*D*) should be adjacent to *Ac* and *p1* sequences. Therefore we used *Ac* casting [36], [37] and inverse PCR to isolate the sequences at the junction of the two duplication segments (Text S1). Comparison with the maize B73 genome sequence (Release 5b.60) [14] indicates that the breakpoints in *P1-rr-T1* and *P1-rr-T481* are located ∼460 kb and ∼5.3 Mb proximal to *p1*, respectively. For each allele we designed two new primers (1 and 2, Figure 1) flanking the predicted insertion sites and used these in PCR together with *Ac*-specific primer Ac5. Primers 1+2 amplified products containing the intact insertion sites, and primers 1+Ac5 amplified the duplication junctions of sequence *a* with 5′ *Ac* (Figure 3); the results indicate that the breakpoint sequence is duplicated in both *P1-rr-T1* and *P1-rr-T481*. Previous semi-quantitative PCR analysis indicated that the *p1* sequence proximal to *Ac* is duplicated; hence these alleles carry duplications. To determine the relative orientations of the duplicated segments, we performed PCR with primers 1+3 which flank the duplication junction of each allele. As shown in Figure 1*D*, primers 1 and 3 are separated by a 4565 bp *Ac* element at the duplication. By use of short PCR cycle times we could preferentially amplify products derived from somatic excision of *Ac*. PCR bands with sizes expected from *Ac* excision were amplified from both *P1-rr-T1* and *P1-rr-T481*; sequencing of the PCR products shows that the sequence *a* of each breakpoint allele is linked to *p1* gene sequences via a short footprint sequence typical of an *Ac* excision (Figure S1), and that the duplicated segments are in direct orientation as shown in Figure 1*D*. Together these results confirm the conclusion that *P1-rr-T1* and *P1-rr-T481* each carry a large segmental duplication of the sequence proximal to *p1*, in direct orientation.

### The white twin *p1-ww-T1* carries a reciprocal deletion

**Figure 4:**
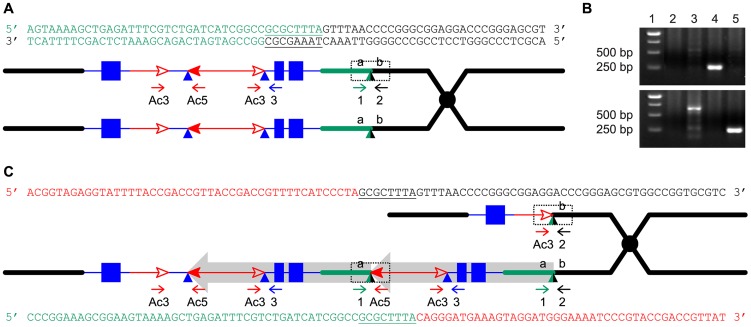
Breakpoint sequences of reciprocal duplication/deletion alleles *P1-rr-T1* and *p1-ww-T1* generated by Reversed Ends Transposition. (*A*) Diagram shows the structure of the progenitor *P1-ovov454* allele prior to RET. Two sister chromatids are shown, with symbols as in Figure 1. The dotted box shows the a/b target site region, whose sequence is indicated above. The color of the letters in the sequences matches the chromatid line color. (*B*) PCR amplification of the deletion/duplication breakpoints in *p1-ww-T1* and *P1-rr-T1* with primers 2+Ac3 (upper panel) or 1+Ac5 (lower panel). Lane 1: DNA ladder, lane 2: *p1-ww[4Co63]*, lane 3: *P1-ovov454/p1-ww[4Co63]*, lane 4: *p1-ww-T1/p1-ww[4Co63]*, lane 5: *P1-rr-T1/p1-ww[4Co63]*. (*C*) Sister chromatid structures of *p1-ww-T1* (upper) and *P1-rr-T1* (lower). Sequences of the deletion and duplication breakpoints (dotted boxes) are shown in color matching the chromatid line color. Note that each breakpoint has a copy of the 8 bp TSD GCGCTTTA which is present in a single copy at the a/b target site in the progenitor allele.

Another prediction of the RET model (Figure 1) is that the white twin alleles (*p1-ww-T1* and *p1-ww-T481*) should each carry a deletion as the reciprocal product of their corresponding red duplication twins. To test this, PCR analysis was performed with primer pairs 2+Ac3 and 1+Ac5 which are specific for the predicted deletion and duplication junctions, respectively (Figure 4). Products of the expected sizes were amplified from *p1-ww-T1* and *P1-RR-T1* (Figure 4B). Importantly, sequencing of the PCR products showed that the 8 bp sequences immediately flanking the *fAc* 3′ end in *p1-ww-T1* and the *Ac* 5′ end in *P1-rr-T1* are identical, indicating their origin as a target site duplication (Figure 4C), the hallmark of *Ac*/*Ds* transposition. This result confirms that the twinned duplication/deletion alleles *P1-rr-T1* and *p1-ww-T1* originated as reciprocal products of a single reversed *Ac* ends transposition event.

We attempted to isolate the *p1-ww-T481* allele, but none of the plants grown from the seven kernels within the white twin sector carried the expected deletion; all carried a standard *p1-ww* allele derived from the normal homologous chromosome. Because the duplication in the corresponding red twin is 5.3 Mb, and a deletion of this size is most likely gametophyte lethal, we suspect that female gametophytes that received the deletion chromosome in meiosis had aborted and thus were not represented in the mature sector. This idea is consistent with the fact that the white sector contained fewer kernels than its red co-twin (*P1-rr-T481*; Figure 2)

**Figure 5:**
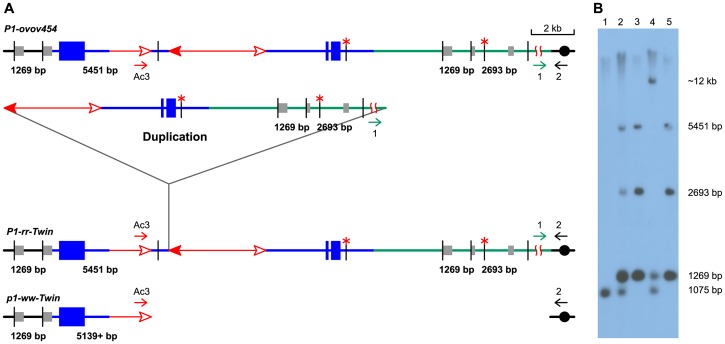
DNA gel blot analysis of the twinned alleles. (*A*) Structure of progenitor *P1-ovov454* allele (upper), and predicted structures of the *P1-rr-Twin* (duplication) and *p1-ww-Twin* (deletion) alleles (lower) generated via reversed *Ac* ends transposition. Blue lines and boxes indicate *p1* sequences, green lines indicate sequences proximal to *p1*, and gray boxes indicate sequences homologous to probe 15. The short vertical black lines indicate *Sal*I sites, and red asterisks (*) mark methylated *Sal*I sites. The other symbols have the same meaning as in Figure 1. (*B*) DNA gel blot. Genomic DNA was digested with *Sal*I and hybridized with *p1* genomic probe 15 (gray boxes in Figure 5*A*). Lane 1: *p1-ww[4Co63]*, Lane 2: *P1-ovov454*/*p1-ww[4Co63]*, Lane 3: *P1-rr-T1*, Lane 4: *p1-ww-T1/p1-ww[4Co63]*, Lane 5: *P1-rr-T481*.

DNA gel blotting was conducted to further test the structures of the candidate duplication alleles (Figure 5). Genomic DNAs were digested with *Sal*I, and the blot was hybridized with *p1*-specific probe 15. The progenitor allele *P1-ovov454* shows three probe 15-hybridizing bands: a 5451 bp band containing *fAc*, a 2693 bp band located proximal to *Ac*, and a 1269 bp band which is present on both sides flanking *p1* and hence has a two-fold intensity on the blot. In the *P1-rr-T1* and *P1-rr-T481* samples, the 2693 bp band is twice the intensity of the 5451 bp band, consistent with a duplication of this proximal segment. In the *p1-ww-T1* lane the 2693 band is deleted, and the 5451 bp band is absent and has shifted to a new band of ∼12 kb due to the deletion. An additional band of 1075 bp present in the *P1-ovov454* and *p1-ww-T1* lanes is derived from the *p1-ww* allele that is present in heterozygous condition in these samples (Figure 5).

### Isolation of additional duplication alleles

As described above, the *P1-rr-T1* and *P1-rr-T481* duplication alleles were isolated from twin sectors with a pericarp phenotype distinct from the parental allele. Multikernel twin sectors are produced by transpositions that occur during a narrow window of early ear development and thus are relatively rare. Therefore we asked whether additional duplication alleles could be isolated from whole ears that exhibited a similar phenotype as that of the red co-twins (i.e. less red/white pericarp variegation). These whole-ear cases could have originated from reversed-ends transposition events that occurred either earlier in embryo development (such that the red twin sector encompassed the entire ear), or as pre-meiotic events. Approximately ∼80 ears of this type were identified among the ∼2000 *p1-ovov454/p1-ww* ears screened. Plants grown from these whole-ear cases were analyzed by semi-quantitative PCR (Figure S2) to detect changes in copy number of the *p1*-proximal sequences. In this way we identified 13 additional candidate duplication alleles. The breakpoints of 11 duplication candidates were cloned via *Ac* casting or inverse PCR (iPCR); sequencing the PCR products revealed that the breakpoints were located at various sites up to 3.3 Mb proximal to the *p1* gene on chromosome 1 (Text S1). Based on the breakpoint sequences and the maize genome sequences, new primers 1 and 2 specific for each candidate allele were designed and used in PCR together with *Ac* primer Ac5. The results of PCR using primers 1+2+Ac5 (Figure S3) confirmed that seven of the 11 candidates carried tandem direct duplications ranging in size from 8157 bp to 3.3 Mb (Table 1). PCR using primers 1+3 flanking the presumed duplication breakpoint confirm that all of the seven alleles derived from whole ears contain tandem direct duplications. The structures of the other four alleles are more complex and are under further investigation.

**Table 1:** The lengths of the duplications.

Alleles	Distance
*P1-rr-E20*	8157 bp
*P1-rr-E45*	203 kb
*P1-rr-E43*	332 kb
*P1-rr-T1*	460 kb
*P1-rr-E317*	459 kb
*P1-rr-E3*	802 kb
*P1-rr-E70*	1.0 Mb
*P1-rr-E10*	3.3 Mb
*P1-rr-T481*	5.3 Mb

These seven candidate duplication alleles were also subject to DNA gel blot analysis (Figure S4); the results show a higher relative intensity of the 2693 bp fragment in all of the candidate alleles except for *P1-rr-E20*, whose 8157 bp duplication does not extend into the 2693 bp fragment detected by the probe. Together the DNA gel blot results confirm the allele structures predicted from the duplication breakpoint sequences. The DNA gel blot results and semi-quantitative PCR indicated that *P1-rr-E301* and *P1-rr-E336* also contain duplications, but their breakpoints are not yet cloned.

### Identification of transposon-induced duplications in the maize genome

**Figure 6:**
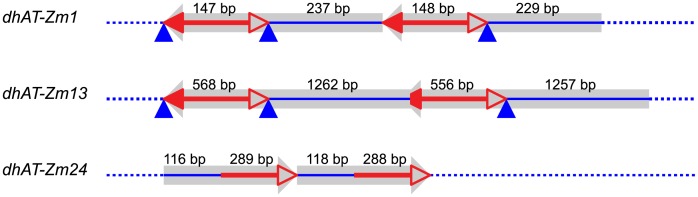
Tandem direct duplications in maize generated by Reversed-Ends Transposition. Red lines with arrowhead(s) indicate the *dhAT* family elements associated with each duplication; solid and open arrowheads indicate the transposon 5′ and 3′ ends, respectively. The truncated solid arrowhead in *dhAT-Zm13* indicates a deletion of 12 bp from the 5′ TIR. The blue lines represent duplicated segments. The blue triangles indicate the transposon target site duplications. Numbers above each line indicate the length of that segment. Sequences and genomic positions are shown in Text S2.

The experiments described above identified nine tandem direct duplication alleles apparently generated *de novo* by RET of *Ac/Ds* elements. If this mechanism has contributed to genome evolution, one would expect to find evidence of transposon-induced duplications in the maize genome sequence. Therefore we conducted a bioinformatics search of the maize B73 reference genome for duplications with the structural features predicted by the RET model. First we identified sequences flanking known *hAT* family transposons and compared the flanking sequences to detect duplications; we then analyzed these candidate duplications for the sequence features predicted by the RET model. In total, 26 known maize *hAT* family transposons, including *Ac/Ds* element and 25 *dhAT* family elements identified in the lab of Dr. Jinsheng Lai, China Agricultural University (personal communication), were used to search for associated duplications in maize B73 reference genome (ZmB73_RefGen_V2). In this way, we identified three small duplicated segments (Figure 6) that have the sequence features predicted by the RET model (Figure 1). These three tandem duplications are associated with 3 different *dhAT* family elements, *dhAT*-Zm1, *dhAT*-Zm13 and *dhAT*-Zm24. The first duplication is located on chromosome 1 and contains two tandem direct repeats of 147 bp and 148 bp that are 93% identical. The duplicated segments are initiated by two *dhAT*-Zm1 elements with 95% sequence identity (Figure 6). The second duplication is located on chromosome 7 and contains two tandem direct repeats of 1262 bp and 1257 bp that are 96% identical. The duplicated segments are initiated by two *dhAT*-Zm13 elements with 95% sequence identity; one is intact (568 bp) and the other has a deletion of 12 bp from the 5′ TIR sequence (Figure 6). In both duplications, the first *dhAT* element is flanked by 8 bp direct repeats that represent the Target Site Duplications (TSDs) generated by *hAT* element insertion. Whereas, the second *hAT* element is flanked on one side by the same TSD as the first element, but the other terminus does not have a matching TSD. This is exactly the structure predicted by the RET model (Figure 1) and observed in the *Ac*-induced duplications (Figure 4): the first transposon has TSDs derived from the original insertion of the transposon (pre-duplication); the second transposon copy has the same TSD on one end, but the other end has a non-matching flanking sequence because it represents the subsequent RET event that generated the duplication. The third duplication (on chromosome 6) has a somewhat different structure, but is still consistent with the predictions of the RET model. This case contains direct repeats of 116 bp and 118 bp that are 99% identical; these repeats are initiated by two fractured *dhAT*-Zm24 elements with 96% identity. The intact *dhAT*-Zm24 element is 904 bp long, whereas these fractured elements contain only 288 bp and 289 bp from the 3′ end. A duplication with these structural features could also be formed by a mechanism of RET as shown in Figure S5 (Movie S2).

## Discussion

By taking advantage of a visual screen to identify chromosome rearrangements associated with *Ac* transposition events, we have isolated and characterized nine tandem duplications that arose *de novo* from a single progenitor allele. The endpoints of all nine duplications coincide precisely with *Ac* termini. Two duplications were isolated from phenotypic twinned sectors, and in one case we were able to recover and characterize a complementary deletion allele. Importantly, the endpoints of the twinned duplication/deletion alleles share a matching 8 bp TSD which is a hallmark of *Ac* transposition. These results indicate that the duplications originated through reversed *Ac* ends transpositions (RET) that occurred during or shortly after DNA replication; the excised *Ac*/*fAc* ends inserted into sites in the sister chromatid, resulting in reciprocal chromatids, one containing a tandem direct duplication, and the other bearing a corresponding deletion (Figure 1). These structures are not consistent with origin via other mechanisms. BIR and FoSTeS generally do not produce a deletion and a reciprocal duplication in the same event [19]. NAHR can generate a deletion and a reciprocal duplication. However, if these duplications were generated by NAHR between non-allelic *Ac* elements, then they should contain three copies of *Ac* (one *Ac* flanking the proximal and distal duplication endpoints, and one between the duplicated segments). All of the duplications we isolated lack an *Ac* element at one breakpoint. Although it is formally possible that one *Ac* element excised after the formation of the duplication, this can be excluded because the sequences at the junctions do not contain any evidence of an *Ac* excision footprint. Moreover, duplications generated via NAHR are recurrent; independent NAHR events between the same repeats generate duplications of the same size. However, our duplications share only one breakpoint in intron 2 of the *p1* gene; the second breakpoint is different for each of the duplications, resulting in a set of nine overlapping duplications ranging in size from 8157 bp to ∼5.3 Mb.

The Drosophila *P* element transposon can undergo various types of alternative transposition events that can produce a multitude of rearrangement structures, depending on which transposon termini are involved in the transposition reactions, and the location of the target site (see [25] for review). In the case of the maize *Ac/Ds* system, fewer types of alternative transposition can occur because the transposition competence of each *Ac/Ds* end is dependent on strand-specific hemi-methylation of the transposon TIR. The tandem duplications described here are entirely consistent with the RET model shown in Figure 1, and with the known restriction on transposition competence of *Ac/Ds* elements [38], [39].

NAHR is reported to occur at a frequency of 10^−5^ to 10^−6^ in human [40]–[42]; in *Arabidopsis*, a frequency of 10^−4^ to 10^−6^ was observed for NAHR between two ∼1.2 kb repeats separated by ∼4.0 kb unique DNA sequence [43]. Rates of NAHR have not, to our knowledge, been reported for maize. Our results indicate that transposition-induced duplications can occur at a relatively high frequency, depending on the presence of an active transposon system with appropriately positioned elements. From a population of approximately 2000 plants, we identified seven whole ears and two twinned-sector ears with transposition-generated tandem direct duplications. DNA gel blotting and semi-quantitative PCR results indicate that two additional cases (*P1-rr-E301* and *P1-rr-E336*; Figure S4) also carry duplications, although we could not clone their breakpoints. The calculated duplication frequency (∼0.5%) is very likely an underestimate for two reasons. First, the visual phenotype used to detect duplications (darker red pericarp and fewer purple aleurone spots) is somewhat subtle and some events may have been overlooked. Second, the screen would not have detected distal duplications because these would not alter the *p1* gene or *Ac* dose. Distal duplications would result from insertion of the excised *Ac/fAc* termini into a site between the *p1* gene and telomere (Figure S5; Movie S2), and these would be expected to occur as frequently as proximal duplications. Thus the real frequency of duplications derived from the *P1-ovov454* allele may be closer to 1%. Given this high frequency, we asked whether *Ac/Ds*-induced tandem duplications could be detected in the maize B73 genome, which contains ∼50 *Ac*/*Ds* elements [44]. However, we failed to find any *Ac/Ds* copies adjacent to a tandem duplication, possibly because the *Ac*/*Ds* elements in the B73 genome are widely separated, and efficient reversed-ends *Ac/Ds* transposition requires two elements in close proximity and correct orientation [29].

In addition to the Drosophila *P* element and *Ac/Ds* systems, the Antirhinnum *Tam3* element, a founding member of the *hAT* transposon superfamily, is known to induce flanking genome rearrangements [45]–[47], possibly via alternative transposition mechanism(s). This suggested that other transposons, in particular *hAT* family elements, may be capable of undergoing alternative transposition to mediate genomic rearrangements. Therefore we extended our bioinformatics searches for transposon-associated tandem duplications to a set of 25 other *hAT* family elements previously identified in the maize B73 reference genome (personal communication). These searches returned a total of 7611 *hAT* element insertions, and among these we identified three tandem direct duplications with the key structural features predicted by the RET model: First, they have exactly two repeated copies, and each repeat is initiated precisely by the transposon. Moreover, in two of the duplications the first *hAT* element is flanked by 8 bp TSDs, while the second (middle) element is flanked by only one of these 8 bp sequences. These features are not expected from other duplication mechanisms such as NAHR, BIR and FoSTeS, but they are perfectly predicted by the RET model. Although the duplications observed are relatively short and their frequency is low, it is possible that some examples may not have been detected for various reasons. First, the maize B73 reference genome sequence still has numerous gaps and uncertainties in the order and orientations of many sequence contigs, and these ambiguities will interfere with the identification of duplications, especially larger ones. Second, those more recent and therefore nearly identical duplications may be under-represented in the reference sequence due to collapse during sequence assembly [48], [49]. Third, those duplications in which either one of the TEs excised after formation of the duplication would not be detected by our search criteria. Nevertheless, we conclude from these results that RET-induced tandem duplication has occurred in maize evolutionary history. Given the high frequency and diversity of Class II transposons present in many eukaryotic species [50], [51], the impact of this mechanism in eukaryotic genome evolution may be significant. The RET model described here provides the conceptual basis for additional bioinformatics searches that will be necessary to assess the actual impact of this mechanism in different species.

## Methods

### Genetic stocks

The maize *p1* gene encodes a Myb-like transcription factor controlling the pigmentation of floral tissues, including kernel pericarp (seed coat) and cob. The suffix of a *p1* allele indicates its expression pattern in pericarp and cob, e.g., *P1*-*rr* specifies red pericarp and red cob, *p1*-*ww* specifies white (colorless) pericarp white (colorless) cob, and *P1*-*ovov* specifies orange variegated pericarp (seed coat) and orange variegated cob. The numeral following the suffix indicates the origin of the allele; alleles with the same phenotype but different numeral may have different structures. The *P1-ovov454* allele conditions a high frequency of colorless sectors, presumably resulting from alternative transposition events which interrupt or delete the *p1* gene [52]. The *p1-ww-[4Co63]* allele is from the maize inbred line 4Co63 [53]; heterozygous plants of genotype *P1-ovov454/p1-ww-[4Co63]* were fertilized with pollen from plants of genotype *C1, r1-m3::Ds [4Co63]*. *Ac* induces excision of *Ds* from *r1-m3::Ds*, resulting in restoration of *r1* gene function and purple aleurone sectors. *Ac/Ds* transposition is subject to the negative *Ac* dosage effect [34], [35], in which increases in *Ac* copy number result in a developmental delay in *Ac/Ds* transposition. If reversed *Ac* ends transposition occurs as shown in Figure 1, two different sister chromatids would be produced: one carrying a tandem direct duplication, and the other a reciprocal deletion (Figure 1*D*). These chromatids will separate into two adjacent daughter cells at mitosis, forming a twinned sector after successive rounds of cell division. The sector with the deletion chromosome has lost *Ac* and exons 1 and 2 of the *p1* gene, and thus should have colorless pericarp with no purple aleurone sectors. The sector with the duplication chromosome retains a functional *P1-ovov454* gene and two copies of *Ac*, and thus should exhibit fewer colorless pericarp sectors, and smaller kernel aleurone sectors.

### Genomic DNA extractions, Southern blot hybridization

Total genomic DNA was extracted using a modified cetyltrimethylammonium bromide (CTAB) extraction protocol [54]. Agarose gel electrophoresis and Southern hybridizations were performed according to Sambrook et al [55] , except hybridization buffers contained 250 mM NaHPO4, pH 7.2, 7% SDS, and wash buffers contained 20 mM NaHPO4, pH 7.2, 1% SDS.

### PCR amplifications

Sequences of oligonucleotide primers used in PCR reactions are given in Table 2; note that primers 1 and 2 are specific to each allele. PCR was performed using HotMaster Taq polymerase from 5 PRIME (Hamburg, Germany). Reactions were heated at 94°C for 2 min, and then cycled 35 times at 94°C for 20 s, 60°C for 10 s, and 65°C for 1 min per 1 kb length of expected PCR product, then 65°C for 8 min. For difficult templates, 0.5–1 M betaine and 4%–8% DMSO were added. The band amplified was purified from an agarose gel and sequenced directly. Sequencing was done by the DNA Synthesis and Sequencing Facility, Iowa State University, Ames, Iowa, United States. *Ac* casting and inverse PCR were performed as described previously [36].

**Table 2:** PCR primers.

Primer 1	*P1-rr-E20*	TAGATTTCCGTTCTTCGTGTGA
	*P1-rr-E45*	GCAACACCTCAAGACCACACGG
	*P1-rr-E43*	GCGTAGCGCACGTCCTCCAC
	*P1-rr-T1*	GCCGAGCGTTCCGTGATCGTGACTC
	*P1-rr-E317*	GTGAAAATCGTGAGGGAGTGGTG
	*P1-rr-E3*	GTCCCACGGCGGCTCGAAGA
	*P1-rr-E70*	GTTGTGAGTGGGAAGTTTCAGGCA
	*P1-rr-E10*	CAGCACTGCTTTGGCGATGTG
	*P1-rr-T481*	ACAGTAGGAGCTTCGCTTACTTCTC
Primer 2	*P1-rr-E20*	GAAAGGTTGTGGAGAATAATAATAAGTAGGGCA
	*P1-rr-E45*	CGCCAAATGTCAGAGGGTAAATC
	*P1-rr-E43*	CGTGCTCGTCAGGTACGCTCG
	*P1-rr-T1*	CGGGACGCATGTGTGTGCTTGAT
	*P1-rr-E317*	CTCCATCCAACGCCCAACTG
	*P1-rr-E3*	CAAACCAGAAAGTTAGCAACCCAG
	*P1-rr-E70*	GGCAGTATGGCACCACGGAG
	*P1-rr-E10*	AAGCCCTGCTCATCGAACGC
	*P1-rr-T481*	CAGTAGACAACGACGAACACAGATG
Ac3		GATTACCGTATTTATCCCGTTCGTTTTC
Primer 3		GCTATCAAACAGGACACGGGAGAGAAT
Ac5		CCCGTTTCCGTTCCGTTTTCGT

### Bioinformatics

The sequences of 26 *hAT* family transposable elements were used as queries to search for homologous elements in the maize B73 reference genome (ZmB73_RefGen_V2) via local BLASTN with default parameters. Two types of homologous sequences were identified: 1) intact elements, which contained both 5′ and 3′ termini; 2) fractured elements, which contained one terminal end (either 5′ or 3′) but having lengths greater than 100 bp. A PERL script was developed to extract two 100 bp segments flanking each transposon, one 5′ adjacent and one 3′ adjacent. Pair-wise comparisons were performed among the segments flanking the same terminal end within each individual *hAT* family. Two *hAT* family members with the same orientation, less than 100 kb apart, and with homologous sequences flanking one terminal end but not the other end were retained for further structural analysis. Such cases were examined manually for the following features: 1) the duplication comprises the complete sequence between the two *hAT* elements, and 2) the duplication is initiated by the transposable element insertion. Sequences that met the above criteria were considered as putative duplications generated by alternative transposition and were examined further for the presence of TSDs as described in the text.

## Supporting Information

Figure S1 **Determining the orientation of duplications by PCR and sequence analysis.** (*A*) Excision of *Ac* from the duplication chromosome brings primers 1 and 3 into close proximity, enabling the amplification of PCR products containing the excision site and footprint (indicated by ×). (*B*) Sequencing chromatograms of *Ac* excision products from *P1-rr-T1* (upper) and *P1-rr-T481* (lower). *Ac* excision is commonly accompanied by minor sequence changes (footprints); the most common *Ac/Ds* footprints in plants are small (1–2) base substitutions or deletions. Genomic DNA prepared from leaf tissue of a single plant may contain molecules from multiple independent somatic *Ac* excision events; these can be detected by direct sequencing of the PCR products (amplified using primers 1 and 3), resulting in multiple peaks beginning at the excision site as seen in the upper chromatogram. The sequences flanking the footprint are identical in different PCR products, hence it is often possible to infer the sequences of the individual major products. The signals giving rise to multiple peaks at each position are recorded in italic letters to aid in interpreting the figure. In *P1-rr-T1*, primer 3 was the sequencing primer, and two types of footprints were identified. In *P1-rr-T481*, primer 1 was the sequencing primer, and two types of footprints are indicated in italics. The presence of a third footprint species can be inferred from the observation of three distinct peaks (A, T and G) at position 81; this third species would be identical to one of the two sequences shown in italics, except for the base at position 81.
(TIF)

Figure S2 **Semi-quantitative PCR of duplication alleles in comparison with parental *P1-ovov454*.** (*A*) Structure of *p1* (blue) and *p2* (green) genes in a tandem duplication allele. The *p2* gene is a paralog of *p1* located ∼70 kb proximal to *p1* in *P1-ovov454* and its derivatives. The boxes indicate exons of *p1*/*p2*, and the blue and green bars under the gene structure indicate the approximate positions of the PCR products. Other symbols have the same meaning as in Figure 1. (*B*) PCR gel. The primer pair used here amplifies a 343 bp band from *p1* and a 420 bp band from *p2*. By comparing the *p2/p1* product band intensities, the *p2/p1* gene ratio of each genotype was estimated and is indicated at the bottom (note that the smaller *p1* band appears to amplify somewhat more efficiently than the *p2* band, hence the product intensity ratio is not identical with the inferred gene copy ratio). The 4Co63 inbred line (lane 1) contains a *p2* gene but lacks *p1*; its *p2/p1* ratio is indicated as 2/0. The progenitor allele *P1-ovov454* and all its derivatives are heterozygous with 4Co63. For *P1-ovov454* (lane 2), the ratio of *p2*/*p1* is 2/1 (two copies of *p2-* one copy from 4Co63 and one copy from the *P1-ovov454* chromosome: one copy of *p1* from the *P1-ovov454* chromosome). Lanes 3–6 are *P1-rr-E43*, *P1-rr-E10*, *P1-rr-E20*, and *P1-rr-E70*, respectively. A *p2/p1* ratio of 2/2 (lane 5) indicates a duplication that does not include *p2* (only *p1* in the *P1-ovov454*-carrying chromosome was duplicated); whereas a 3/2 ratio (lanes 3, 4, 6) indicates a duplication that extends beyond *p2* (both *p1* and *p2* in the *P1-ovov454*-carrying chromosome were duplicated).
(TIF)

Figure S3 **PCR analysis of progenitor *P1-ovov454* and duplication alleles obtained from whole ears.** PCR was performed using primers 1+2+Ac5 (see Figure 1 for primer locations; note that primers 1 and 2 are specific for each allele). Primers 1+2 amplify the transposition target site (present in the progenitor *P1-ovov454* and each duplication allele), while primers 1+Ac5 amplify the duplication junction (present only in the duplication alleles). Lane 1: DNA ladder; lanes 2–4: H_2_O, *P1-ovov454*, *P1-rr-T1*; lanes 5–7: H_2_O, *P1-ovov454*, *P1-rr-T481*; lanes 8–10: H_2_O, *P1-ovov454*, *P1-rr*-*E70*; lanes 11–13: H_2_O, *P1-ovov454*, *P1-rr*-*E3*; lanes 14–16: H_2_O, *P1-ovov454*, *P1-rr*-*E43*; lanes 17–19: H_2_O, *P1-ovov454*, *P1-rr*-*E45*. Six of the nine alleles isolated from whole ears are analyzed here. The other three alleles were not included because they were heterozygous with 4Co63 which can produce a product of the same size as that in *P1-ovov454* using primers 1+2.
(TIF)

Figure S4 **DNA gel blot analysis of progenitor *P1-ovov454* and duplication alleles.** Genomic DNA was digested with *Sal*I and hybridized with genomic probe 15. See Figure 5 for allele structures and probe locations. Lane 1: *p1-ww[4Co63]*; Lane 2: *P1-ovov454*/*p1-ww[4Co63]*; Lane 3: *P1-rr-T1*; Lane 4: *p1-ww-T1/p1-ww[4Co63]*; Lane 5: *P1-rr-T481*; Lane 6: *P1-rr-E10/p1-ww[4Co63]*; Lane 7: *P1-rr-E70/p1-ww[4Co63]*; Lane 8: *P1-rr-E3/p1-ww[4Co63]*; Lane 9: *P1-rr-E317/p1-ww[4Co63]*; Lane 10: *P1-rr-E43/p1-ww[4Co63]*; Lane 11: *P1-rr-E45/p1-ww[4Co63]*; Lane 12: *P1-rr-E20/p1-ww[4Co63]*; Lane 13: *P1-rr-E336/p1-ww[4Co63]*; Lane 14 *P1-rr-E301/p1-ww[4Co63]*
(TIF)

Figure S5 **Reversed *Ac* ends transposition generates distal direct duplication.** The two lines indicate sister chromatids joined at the centromere (black circle). All the symbols have the same meaning as in Figure 1 except the green line now indicates the *p1*-distal segment. (*A*) *Ac* transposase cleaves the lower chromatid at the 3′ end of *fAc* and the 5′ end of *Ac*. (*B*) Following transposase cleavage, the inter-transposon segment is joined to form a circle. (*C*) Transposon ends insert into the sister chromatid at a distal site. The 3′ end of *fAc* joins to the green segment (b) to generate a distal direct duplication, while the 5′ *Ac* end joins to the black segment (a) to generate a distal deletion. Note that both chromatids carry one copy of *Ac* element. For animation, please see Movie S2.
(TIF)

Movie S1 **Reversed *Ac* ends transposition generates direct duplication.** The animation can be played in Internet Explorer or Firefox. The buttons at the bottom are *Play*, *Fast Forward*, *Rewind*, and *Pause*. The two lines indicate sister chromatids of maize chromosome 1, joined at the centromere (black). The blue boxes are exons 3, 2, and 1 (left to right) of the *p1* gene. Red lines with arrowhead(s) indicate *Ac/fAc* insertions, and the open and solid arrowheads indicate the 3′ and 5′ ends, respectively, of *Ac*/*fAc*. The two blue ovals indicate *Ac* transposase, which cleaves the lower chromatid at the 3′ end of *fAc* and the 5′ end of *Ac*. Following transposase cleavage, the internal *p1* genomic sequences are joined to form a circle. Then the *Ac/fAc* transposon ends insert into the upper sister chromatid at a proximal target site (a/b) to generate a proximal deletion (upper chromatid) and a direct duplication (lower chromatid).
(SWF)

Movie S2 **Reversed *Ac* ends transposition generates distal direct duplication.** All the symbols have the same meaning as in Movie S1 except the green line now indicates the *p1*-distal segment. The animation can be played in Internet Explorer or Firefox. The buttons at the bottom are *Play*, *Fast Forward*, *Rewind*, and *Pause*. The two lines indicate sister chromatids of maize chromosome 1, joined at the centromere (black). The blue boxes are exons 3, 2, and 1 (left to right) of the *p1* gene. Red lines with arrowhead(s) indicate *Ac/fAc* insertions, and the open and solid arrowheads indicate the 3′ and 5′ ends, respectively, of *Ac*/*fAc*. The two blue ovals indicate *Ac* transposase, which cleaves the lower chromatid at the 3′ end of *fAc* and the 5′ end of *Ac*. Following transposase cleavage, the internal *p1* genomic sequences are joined to form a circle. Then the *Ac/fAc* transposon ends insert into the upper sister chromatid at a distal target site (a/b) to generate a distal deletion (upper chromatid) and a direct duplication (lower chromatid).
(SWF)

Text S1 Breakpoint sequences of *Ac*-induced duplication/deletion alleles. *Ac* sequences are shown in red text. Duplication breakpoint sequences are shown in green text, and the deletion breakpoint sequence is shown in black text. The eight bp Target Site Duplications identified in *P1-rr-T1* and *p1-ww-T1* alleles are highlighted in yellow.
(DOC)

Text S2 Sequences are shown for tandem direct duplications associated with *dhAT-Zm1*, *dhAT-Zm13*, and *dhAT-Zm24*. The headings indicate the maize chromosome number and sequence position of each duplication. Duplicated segments are indicated by blue text and gray shading; transposon sequences are shown in red text, and Target Site Duplications are highlighted in light blue.
(DOC)
