# Supplementary figures and images for: Generation of Tandem Direct Duplications by Reversed-Ends Transposition of Maize Ac Elements
Source: PLoS Genet. 2013 Aug 15;9(8):e1003691. doi: 10.1371/journal.pgen.1003691 (PMC3744419; doi:10.1371/journal.pgen.1003691)

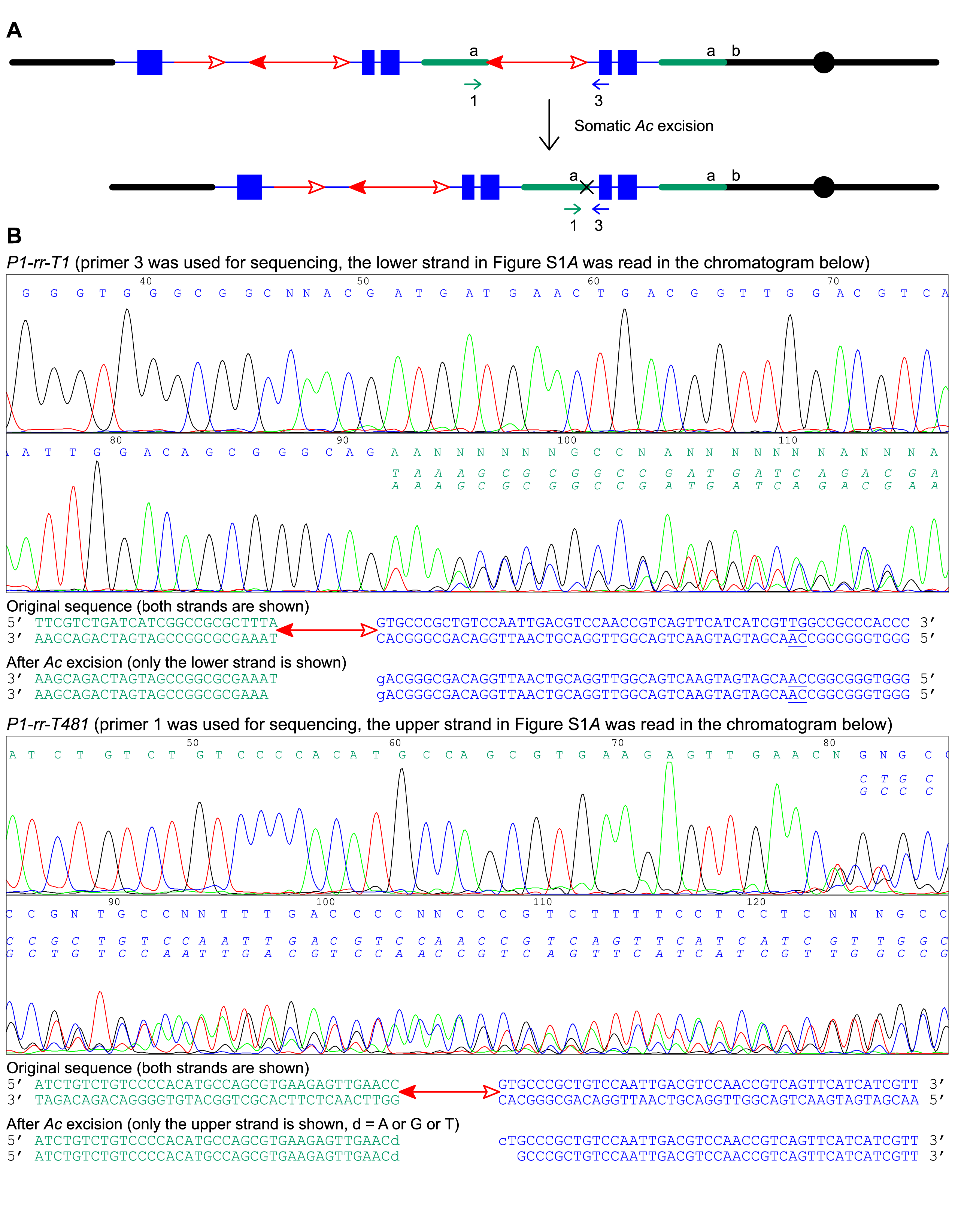

Supplement: Figure S1 — Determining the orientation of duplications by PCR and sequence analysis. (A) Excision of Ac from the duplication chromosome brings primers 1 and 3 into close proximity, enabling the amplification of PCR products containing the excision site and footprint (indicated by ×). (B) Sequencing chromatograms of Ac excision products from P1-rr-T1 (upper) and P1-rr-T481 (lower). Ac excision is commonly accompanied by minor sequence changes (footprints); the most common Ac/Ds footprints in plants are small (1–2) base substitutions or deletions. Genomic DNA prepared from leaf tissue of a single plant may contain molecules from multiple independent somatic Ac excision events; these can be detected by direct sequencing of the PCR products (amplified using primers 1 and 3), resulting in multiple peaks beginning at the excision site as seen in the upper chromatogram. The sequences flanking the footprint are identical in different PCR products, hence it is often possible to infer the sequences of the individual major products. The signals giving rise to multiple peaks at each position are recorded in italic letters to aid in interpreting the figure. In P1-rr-T1, primer 3 was the sequencing primer, and two types of footprints were identified. In P1-rr-T481, primer 1 was the sequencing primer, and two types of footprints are indicated in italics. The presence of a third footprint species can be inferred from the observation of three distinct peaks (A, T and G) at position 81; this third species would be identical to one of the two sequences shown in italics, except for the base at position 81. (TIF) [file pgen.1003691.s001.tif]

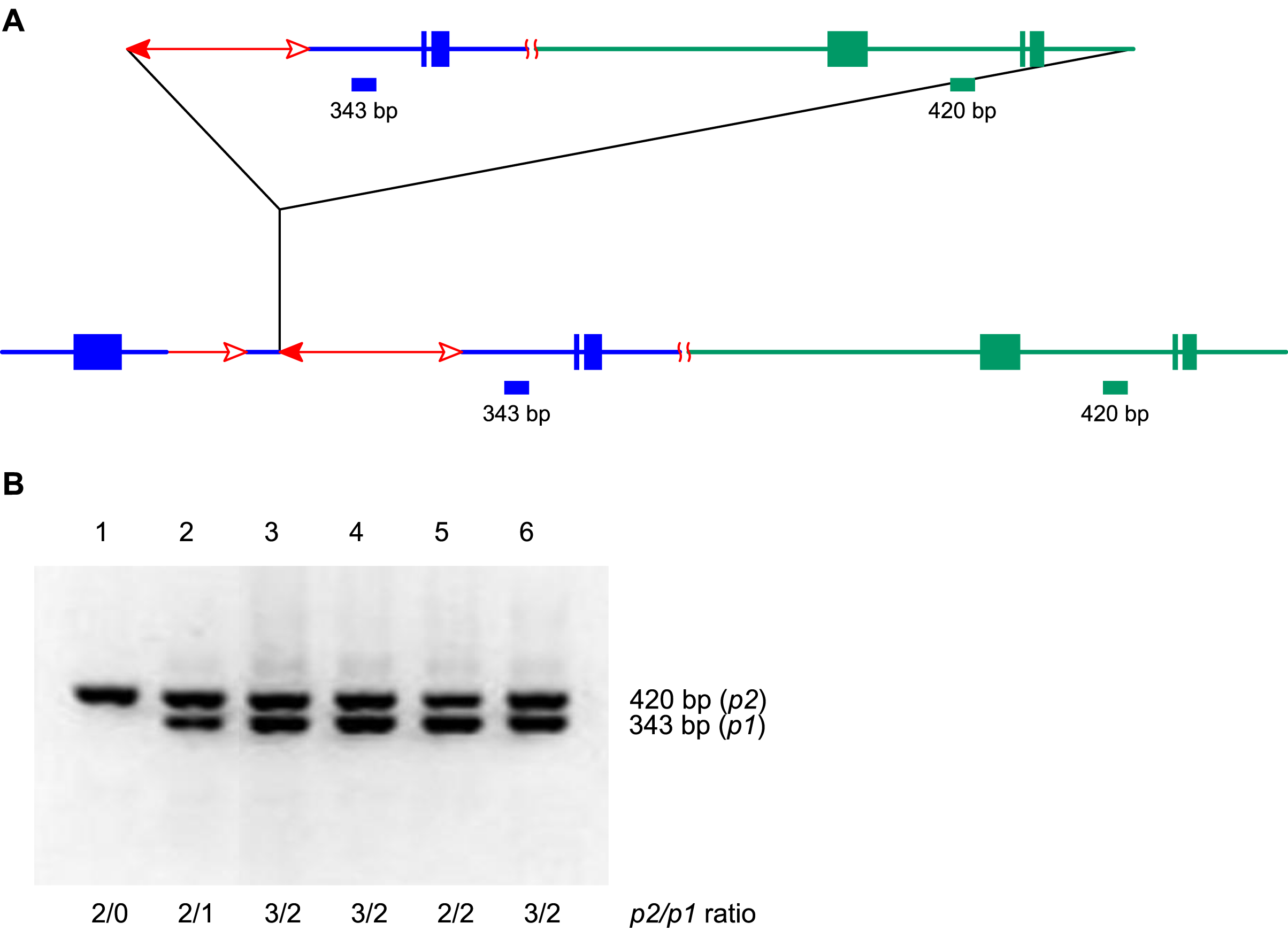

Supplement: Figure S2 — Semi-quantitative PCR of duplication alleles in comparison with parental P1-ovov454. (A) Structure of p1 (blue) and p2 (green) genes in a tandem duplication allele. The p2 gene is a paralog of p1 located ∼70 kb proximal to p1 in P1-ovov454 and its derivatives. The boxes indicate exons of p1/p2, and the blue and green bars under the gene structure indicate the approximate positions of the PCR products. Other symbols have the same meaning as in Figure 1. (B) PCR gel. The primer pair used here amplifies a 343 bp band from p1 and a 420 bp band from p2. By comparing the p2/p1 product band intensities, the p2/p1 gene ratio of each genotype was estimated and is indicated at the bottom (note that the smaller p1 band appears to amplify somewhat more efficiently than the p2 band, hence the product intensity ratio is not identical with the inferred gene copy ratio). The 4Co63 inbred line (lane 1) contains a p2 gene but lacks p1; its p2/p1 ratio is indicated as 2/0. The progenitor allele P1-ovov454 and all its derivatives are heterozygous with 4Co63. For P1-ovov454 (lane 2), the ratio of p2/p1 is 2/1 (two copies of p2- one copy from 4Co63 and one copy from the P1-ovov454 chromosome: one copy of p1 from the P1-ovov454 chromosome). Lanes 3–6 are P1-rr-E43, P1-rr-E10, P1-rr-E20, and P1-rr-E70, respectively. A p2/p1 ratio of 2/2 (lane 5) indicates a duplication that does not include p2 (only p1 in the P1-ovov454-carrying chromosome was duplicated); whereas a 3/2 ratio (lanes 3, 4, 6) indicates a duplication that extends beyond p2 (both p1 and p2 in the P1-ovov454-carrying chromosome were duplicated). (TIF) [file pgen.1003691.s002.tif]

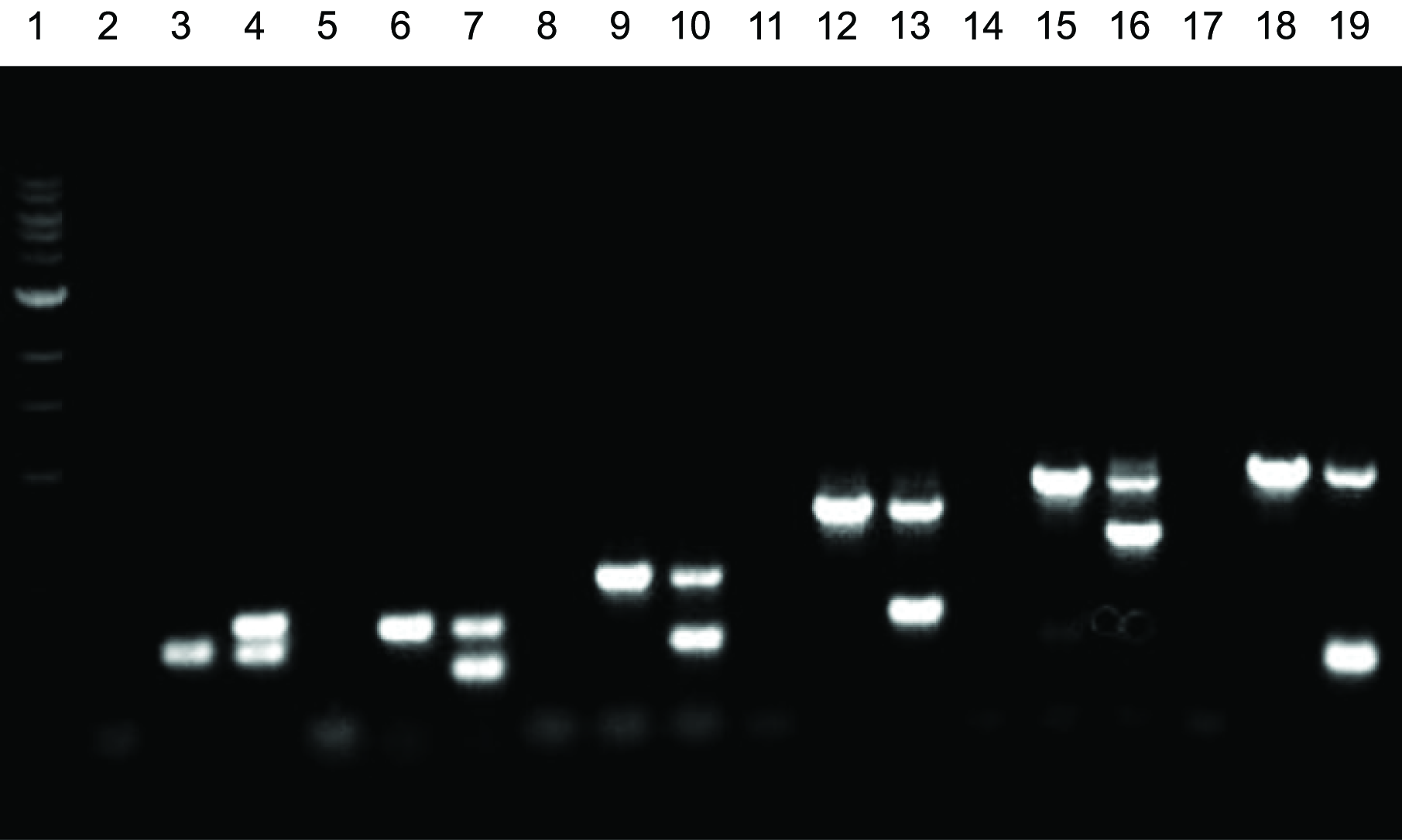

Supplement: Figure S3 — PCR analysis of progenitor P1-ovov454 and duplication alleles obtained from whole ears. PCR was performed using primers 1+2+Ac5 (see Figure 1 for primer locations; note that primers 1 and 2 are specific for each allele). Primers 1+2 amplify the transposition target site (present in the progenitor P1-ovov454 and each duplication allele), while primers 1+Ac5 amplify the duplication junction (present only in the duplication alleles). Lane 1: DNA ladder; lanes 2–4: H2O, P1-ovov454, P1-rr-T1; lanes 5–7: H2O, P1-ovov454, P1-rr-T481; lanes 8–10: H2O, P1-ovov454, P1-rr-E70; lanes 11–13: H2O, P1-ovov454, P1-rr-E3; lanes 14–16: H2O, P1-ovov454, P1-rr-E43; lanes 17–19: H2O, P1-ovov454, P1-rr-E45. Six of the nine alleles isolated from whole ears are analyzed here. The other three alleles were not included because they were heterozygous with 4Co63 which can produce a product of the same size as that in P1-ovov454 using primers 1+2. (TIF) [file pgen.1003691.s003.tif]

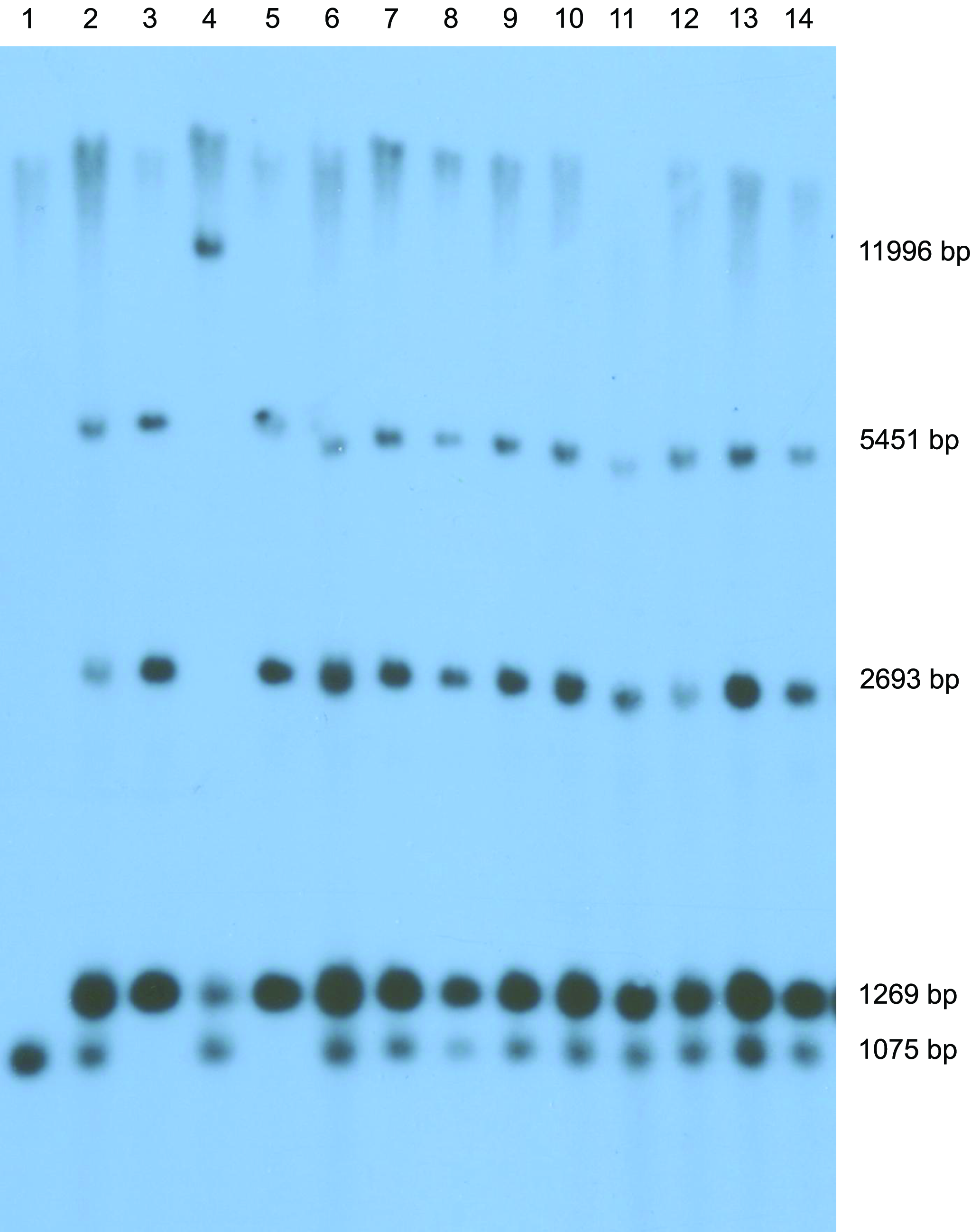

Supplement: Figure S4 — DNA gel blot analysis of progenitor P1-ovov454 and duplication alleles. Genomic DNA was digested with SalI and hybridized with genomic probe 15. See Figure 5 for allele structures and probe locations. Lane 1: p1-ww[4Co63]; Lane 2: P1-ovov454/p1-ww[4Co63]; Lane 3: P1-rr-T1; Lane 4: p1-ww-T1/p1-ww[4Co63]; Lane 5: P1-rr-T481; Lane 6: P1-rr-E10/p1-ww[4Co63]; Lane 7: P1-rr-E70/p1-ww[4Co63]; Lane 8: P1-rr-E3/p1-ww[4Co63]; Lane 9: P1-rr-E317/p1-ww[4Co63]; Lane 10: P1-rr-E43/p1-ww[4Co63]; Lane 11: P1-rr-E45/p1-ww[4Co63]; Lane 12: P1-rr-E20/p1-ww[4Co63]; Lane 13: P1-rr-E336/p1-ww[4Co63]; Lane 14 P1-rr-E301/p1-ww[4Co63] (TIF) [file pgen.1003691.s004.tif]

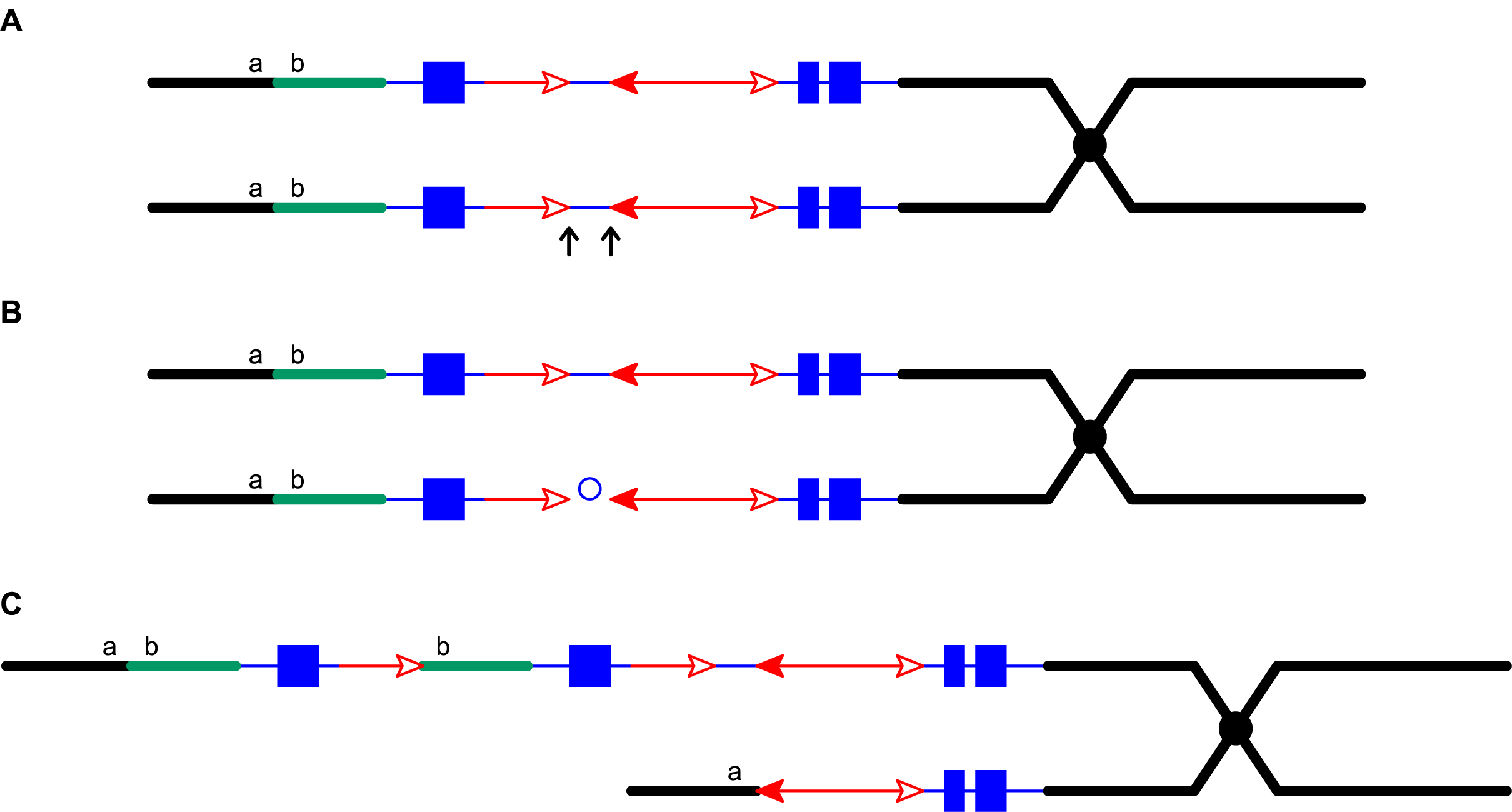

Supplement: Figure S5 — Reversed Ac ends transposition generates distal direct duplication. The two lines indicate sister chromatids joined at the centromere (black circle). All the symbols have the same meaning as in Figure 1 except the green line now indicates the p1-distal segment. (A) Ac transposase cleaves the lower chromatid at the 3′ end of fAc and the 5′ end of Ac. (B) Following transposase cleavage, the inter-transposon segment is joined to form a circle. (C) Transposon ends insert into the sister chromatid at a distal site. The 3′ end of fAc joins to the green segment (b) to generate a distal direct duplication, while the 5′ Ac end joins to the black segment (a) to generate a distal deletion. Note that both chromatids carry one copy of Ac element. For animation, please see Movie S2. (TIF) [file pgen.1003691.s005.tif]
