# Supplementary material for: Generation of Tandem Direct Duplications by Reversed-Ends Transposition of Maize Ac Elements
Source: PLoS Genet. 2013 Aug 15;9(8):e1003691. doi: 10.1371/journal.pgen.1003691 (PMC3744419; doi:10.1371/journal.pgen.1003691)
Supplement: Text S1 — Breakpoint sequences of Ac-induced duplication/deletion alleles. Ac sequences are shown in red text. Duplication breakpoint sequences are shown in green text, and the deletion breakpoint sequence is shown in black text. The eight bp Target Site Duplications identified in P1-rr-T1 and p1-ww-T1 alleles are highlighted in yellow. (DOC) [file pgen.1003691.s008.doc]

**Text S1.**

*P1-rr-T1*

ACTAACAAAATCGGTTATACGATAACGGTCGGTACGGGATTTTCCCATCCTACTTTCATCCCTGTAAAGCGCGGCCGATGATCAGACGAAATCTCAGCTTTTACTTCCGCTTTCCGGGACGGGTACGTCATCGCCGGCTGGATCGGATCGGATCACGCACGCAGCGGCCACTTTGCCCGTGAGTCACGATCACGGAACGCTCGGCA

*p1-ww-T1*

TTTTCGTTTCCGTCCCGCAAGTTAAATATGAAATGAAAACGGTAGAGGTATTTTACCGACCGTTACCGACCGTTTTCATCCCTAGCGCTTTAGTTTAACCCCGGGCGGAGGACCCGGGAGCGTGGCCGGTGCGTCCGACGACCTGTACGTTACGTATTCCACCGCGGAACTACATAAACGTACGGACCCAAACAAAAGCGGCAGATCAAGCACACACATGCGTCCCG

*P1-rr-T481*

AATCGGTTATACGATAACGGTCGGTACGGGATTTTCCCATCCTACTTTCATCCCTGGGTTCAACTCTTCACGCTGGCATGTGGGGACAGACAGATAGGCCTCCCTGTCGCCCAGCTCCTCACTCGTTTCAAAGGTCAGAGAAGTAAG

*P1-rr-E10*

AATCGGTTATACGATAACGGTCGGTACGGGATTTTCCCATCCTACTTTCATCCCTGTGTCCGCCTGCTGCACGCGCGCGCACCATGAGAACAGTGCGCTTCTAGAACTCAGAGACTCAGTCATCTAAATCATGCATATAATTACTCGTAGCAGTGTTGTTGCTTCATCATCAGTGATCACTTAATCGTCTGAGGCGAGAGTAGGGACCATAGTAGCCATTTGCGGTCATGCATGTATGCATCACACTACTGCTCCCGGCCTACTTGGGCATGCATGCACTTGGCACGAATACTTTAGACACAGATCGTACCGAGAAGCTAGCATAGCATATATATGAAGTGGTACTCGGCCATGGCGTGGCAAGCATTAGCCTGCCTCTGCACACACACTGCGAGCTAGCGCCCTAATAAATAAGTTGTTAAGGAGCGAGCATTAGCGTGCCTAGCTATAGCATCTATTAATGCTCTGATTATAGAATACATTGATGACAAGCATGCAGGAGCTAGCTAGCTGCTGGATGAGGTGCTCATCATGATAGGAGATCAACTAGGTTGGTGGCCTCTGGTGAAAACTACTTGTACTACCGTTGCAACATGGATCATGGCTTTGGGCTTTGGCTGACATCGACGCGCCTATAAAATTTTTGATATCTCGGCTACCATGCATGCATGCATATGCATGTGTGGGAGG

*P1-rr-E70*

AATCGGTTATACGATAACGGTCGGTACGGGATTTTCCCATCCTACTTTCATCCCTGAGTAACAGAGAGCAGTCAGCTCGTGCAATTGCGTGTGCACCAACTAGCCTGATTAAATAAGGGGCTTGATCTTTTGTCCGCATTGCCAACAATCGTAGAACCGGGCCGCTATAGGGGGCATGCAGTGAATTTTCCAAAGATGCCTAGGAAACATGCCTGAAACTTCCCACTCACAAC

*P1-rr-E3*

AACTAACAAAATCGGTTATACGATAACGGTCGGTACGGGATTTTCCCATCCTACTTTCATCCCTGCGATGCAGCGACCCCGACGGCGAGCACTTGCTGCTCCGGGCGTTCAAGAACAAGGCGGACTACTGCCGCGTCCACGGCTTCGACATCTTCTACAGCACCGCGGTGCTCGACGCCGAGCTGTCGGGGTTCTGGTCCAAGCTGCCGCTGCTGCGGACGCTGATGCTCGCGCACCCGGAGACGGAGCTCCTGTGGTGGGTGGACTCCGACGTCATCTTCACCGACATGCTCTTCGAGCCGCCGTGGGACAAGTACGCTGCTCACAACCTCGTGCTCCCGGGCTCCGAGGAGAAGGTGTACACCGTTAAGAGCTGGATCGGCATCAAC

*P1-rr-E317*

AATCGGTTATACGATAACGGTCGGTACGGGATTTTCCCATCCTACTTTCATCCCTGGTCTGCCAGGGGGGCAGGGCAGGGCAGGTACCCCTGCCGTGCACGACACACCGTGACAGATCCGCTGCCGGCCGGATCACGGGCGGGCCACCACTCCCTCACGATTTTCACATGGCCTTGCCGGATCCGGCGGGTGATGAAGCGGAACGGGCACGTACTCGCAGTCTCGCG

*P1-rr-E43*

AATCGGTTATACGATAACGGTCGGTACGGGATTTTCCCATCCTACTTTCATCCCTGCCTGCAGTGCTTCGAGGACGAACAGGTAAGCTAACAAGCAAGAGCGTGTTTGGTTTCATGCTAGGACAGAGTTGCATACCACGTAGCTATCATAAGCCTTCCACCACGTAGCTATCACAGCCTGTCGATTTCGTTCGGTCGCCTGACGGTAAACATCGCTGCC

*P1-rr-E45*

AATCGGTTATACGATAACGGTCGGTACGGGATTTTCCCATCCTACTTTCATCCCTGTCTGTAGGGGTCCGGGCCCGTCGATGAATACTGGGGTGTACTCCCTTTCCTTGCCACGTGGCGCTCCTGGACCTGTCCATGCGGTTGGGTTGGGCGCCATTCTCCGTGTGGTCTTGAGGTGTTGCACGGGTGCGGCGCCTTCATCCTGTAGTAGAGGGTACCCCTGATACAGGGTATCGACAAATATAATTTAATGTTATCGATTACTCGTTATGTTACCAATATATAAACCAAACGACACCTAGATGTTACTGGCCAAAGTTGAGTTTGGAGCC

*P1-rr-E20*

AATCGGTTATACGATAACGGTCGGTACGGGATTTTCCCATCCTACTTTCATCCCTGGTCCGCTGCTATATTATGGCCGGCCGTGGCGTGCCCTCTCTAGCCAGCACAGCACACACACTGGAAAGTGCAAGCTGTAGTGAGACCTGCGCGACTGCCAGCGTGTATCCGCGCGGCAAGGAGCGTAGCGCGCGGTCGTCGGCCCGCACGGCCACCAACTCCCTTGGACGCACGCGCGCGCGCGACCAGCTGCTAACCGTGCGCAAGTAGTAGTGCGACTTCGCCGCCGGCCGGGATCGCTAGCTCGATCGATCGGCGGGACCACATACGACTCCG
