# Supplementary material for: Generation of Tandem Direct Duplications by Reversed-Ends Transposition of Maize Ac Elements
Source: PLoS Genet. 2013 Aug 15;9(8):e1003691. doi: 10.1371/journal.pgen.1003691 (PMC3744419; doi:10.1371/journal.pgen.1003691)
Supplement: Text S2 — Sequences are shown for tandem direct duplications associated with dhAT-Zm1, dhAT-Zm13, and dhAT-Zm24. The headings indicate the maize chromosome number and sequence position of each duplication. Duplicated segments are indicated by blue text and gray shading; transposon sequences are shown in red text, and Target Site Duplications are highlighted in light blue. (DOC) [file pgen.1003691.s009.doc]

**Text S2.**

>dhAT-Zm1 chr1 dna chromosome:AGPv2:1:189641847:189640681

TTGGGCTTACTGGTTTAAATGGGGTTTTGTTAAGCAGCTAGACGTATTACTTTTTGGTTGAAGATTGCTCACTTCAAGGATACCTCAATATTGCTAACGCCTAACAGAACAGTCAAAACAATCTATTACAGGAAGTATGTTTACTAGGAATATAGTTCTGAAACACAGTTTGGACGAGTGAACAAAATCTAAATGAGACATATATGTAGGGGTGGTAATAGATCCCGATCCAAATGTTTCTTCACAAAATGGTAGGACTCTAAATAAATTTTAGTTAAAAAATGAATAGAAATAGGACCCGATCCGATCCTTAAATTTTATAGTGTAAAATTTAGAGCCCATTGTCACCCCTACATATATGACACACTCCCCTACCGTTTGTGTTTTATAATGAAGAGCCACTCGGCCACTCCATATCATTTTTGTAATGCACATTTAGATAGCCATTCTTCTTTGTAGCCTACAAAATATTTGGCTGCTGCTTTAAGAATGGAGACATTATACATGTATCAAAATGCTAGAGACTTGTCATTTTGTCTTGTTTTTAGAAATAGGATTGTTCATACTTGAGACTTGTGAATTTCTCAATTTAGGGGTGGTAGTGGATCGCGATCCAAATGCTTATTCACAAAATGGTAGGGCTCTAAATAAATTTTAGTTCAAAAATGAATAAAAAATAGGGCCCGATCCGATCCTTCAATTTTATAGTGTAAAATTTAGAGCCCATTGTCACCCCTACATATATGACACACTCCACTACCGTTTGTGTTTTATACTGAAGAACCACTCCATATCATTTTTGTAATGCACATTTAGATAGCCATTCTTCTTTTTAGCCTACAAAATATTTGGCTGCTGCTTTAAGAATGGAGACATTATACATGTATCATAATGCTAGAGACTTGTCATTTTGTCTTGTTTTTAGAAATAGGATTGCTCATACTTGAGACTTGTGAATTTCTCAATTTCGCAGCAATTTTCTTTATCCAAATATAAAAAAAATTCGACCTGCGAGGGGTAAGACAGCCCCCGGGCATTATATTAAGAAGAAGATCTTCTCACGCGGGTTGAGAAAACCTCCGAATCCTTGCCCCACCCATACACAGCGGCACCGTAGCCCATGTGAGAAACGACCGCGACCGGGGCCGGGCCTTAGACCTGTGCTTT

>dhAT-Zm13 chr7 dna chromosome:AGPv2:7:104415052:104411002

TAAAGGGAGATCAACGACATACAACCATCTTCCTCCACTGGTTCCGTCGCTATTTTCTCCTCAGCCACCCTCCGCGACCGAGTGCCATAGCCGCGGCAGCATGCTGGTCAGAGCTCGCCATCCAAGCCCGAAAAAAAGCCCGGACGCCACTACACAAATCCTACACGGCCACGATGATCCTACGGTCAGAACGCCGCCTACCCATTACTAGTACTGGGAATCTAGGCCGGGCTTTTCGGGCCAGCACGAGCACAGCCCGAAAATAAGAGCCCAAAGCACGGCCTGGCACGAAATAATATGGGCCGGGCTAGCACGGCCCGAAGGCGGGCCTGGGCCGGGCCTCAAATTCTGACCCATCGGGCCCCGGCACAGCCCGCATAGATGGGCCGGGCTTGGGCCAACACAGCCCAATGAAGCCCAATCTATTTAATTTCTTTAATTTAGTAAGATATCGAGTTTATATTGTTGTTATATTTGTAGTTTATATGGTTAAATGATGCTATAATTGTTTAATATCTTTAATTTAGTAAGCTATGAACTTTATATGGTTATAATATTTTCATTTTATGTGGTCAAATATACGGGCCGGGCTTGGGCCGGCACTGCCCAACGAAGGCACGACGTGGTTTAGGGCTGGGCCGGGCCCTGTTTTTACATTTCGAGCTGGCACGGCATGGCCCAAAAATTGTTTGGGCTTTCCTGGCCCGAACTTGTTTGGCATGAAGCACGACGGGCTTGGGCTGGACTGGCCCGGCCTGGCCCAATTCCTAGCACCACCCATTACCCGACAGCATCGGCCGGAGCTGTACATGACTCCAGCATGACAGCTCCATAACCGAACACCCAGGCACACTCCCACGCCGCGGGCACTCCGATTCGACAGGAGCTAAGCATAGCGTGGTTTGGGGCGAGTCCACGGGGCATGGCCATACCAGAGATTAGGCTTCAAGAGGGTTCCTGCCCACAACGCACGTCGATCCGGTGATGAAGTACTTCGGTGAGGTCCTCGGGCGTGTACCCCTGCACGAGCGCCATCAAGTCGGCGACACCGCCGGTGGAGGACGAGGGCACCATAGTGCTCGGCCAGATGATTGGCGATGGCGCAAGGTCTTCGGCCGACAAGATTCTTGGCAGCGCTCTATCTCTCTCTCCGTTGACGCACGCCAGTTCCCCCGTATGATTATGGAAGCAAGGAACCCGGCGGCAGGCTTTCACTATCTCTCAGGCCTCTCTCTCCTCACTAGTTGTAGGCTTAGCAGCAACACCCGAGGTCGGATCCCGTCTGCGGTGTGCCGGGAACATGAGTAGGAACGACGGCTAACAAGAGTCTTCCTCGCCAAAAGGGTGACCAGACTTACATGAGAAGAGGCTGACGCCTTCAGCCCACCAGTCAGCGCCGAGGAGGCCAGGAGTGGGCCGACGTGGAGTATTAGGGGCTGCGTAGTGGGTCCCACGGTCGCTGCAGCGAGCCCAGGCGCGCGCGGTGGGGAGTGAGGCTAAATTTCCAATTTATGCACAAGTTTAATCTCTAAATTTCCAATTTATGCACAAGTAAATCTCCATCATGGGATGCAAAGTTCATTTTATTAATTTATGTTTGACCCTCTAAAGGGTAATATTCGAGCAAGGAGTATTCCTCTCTATTACCTTATTTTTCCGAGTTTAAATTTTTACGTATTAGATTTAAAAAGAAGATCTAGTCCCGACTTTGAGTCATTTATAGTTAATATTCATGTAAATGCTTATAGTGCATCATTTAACAATATGCTTAATCATTTAGTTGAATAGAAAACTTTTCTATTTCTTCTTTTCTAAAAATGGTTCTTAGTTTTCCAAAATAGTTCTTGGCTTTCAAAATTTCGTCCTTAGTTTTCAATACTCAAGTATAATTTGAGATATCTGAATTTACTATTTTATCTCTTCATATATTTATTTTATTATCATTAATAAATTTTTTTCTTACACAGATTTTGGGCCTTACAGCACCTCCATTTCTCACATGTTTTTTCTTAAGTAGAAGATCTCTGTTAGGGGGAGTTTAGGCCGGGCTTTTCGGGCCAGCACGAGCACAGCCCGAAAATAAGAGCCCAAAGCACGACCCGACACGAAATAATATGGGCCGGGCTAGCACGGCCCGAAGGCGGGTCTGGGCCAGGCCTCAAATTCCGACCCATCGGGCCCCCGGCACAGCCCGCATAGATGGGCCGGGCTTGGGCCGGCACAGCCTAATGAAGCCCAATCTATTTAATTTCTTTAATTTAGTAAGATATCGAGTTTATATTGTTGTTATATTTGGAGTTTATGTAGTTAAATGATGTTATAATTGTTTAATATCTTTAATTTAGTAAGCTATGAACTTTATATGGTTATAATATTTTCATTTTATGTGGTCAAATATACGGGCCGGGCTTGGGCCGGCACTGCCCAACGAAGGCACGACGTGGTTTAGGGTTGGGCCGGGCCCTATTTTTACACTTCGGGCTGGCACGGCATGACCCAACAATTGTTTGGGCTTTTCTAGCCCGAACCTGTTTGACACGAAGCACGACAGGCTTGGGCTGGGCTGGCCCGGCCTGGCCCAATTCCCAGCACCACCCATTACCCGACAACATCGGCCAGAGTTGTACATGACTCCAGCATGACGGCTCCATAACCGAACACCCAGGCACACTCCCACGCCGCGGGCACTCCGATTCGACAGGAGCTAAGCATAGCGTGGTTTGGGGCGAGTCCATGGGGCATGGCCATACCAGAGATTAGGCTTCAAGAGGGTTCCTGCCCACAGTGCACGTCGATCCGGTGATGAAGTACTTCGATGAGGTCCTCAGGCGTGTACCCCTGCACGAGCGCCATCAGGTCGGCGGTGCCGCTGGTGGAGGACGAGGGCACCATAGTGCTCGGCCAGATGATAGGTGATGGCGCAAGGTCTTCGGCCGACAGGATTCTTGGCAGCGCTCTCTCTCTCTCTCCGTTGACGCACGCCAGTTCCCCCGTATGATTATGGAAGCAAGGAACCCGGCGGCAGGCTTTCACTATCTCTCAGGCCTCTCTCTCTCCTCACTGGTTGCAGGCGCAGCAGCAACACCCAAGGTCGGATCCCGTCCGCGGTGTGCCGGGAACATGAGTAGGAACGACAACTAACAAGAGTCTTCCTCGCCAAAAGGGCGACCAGACTTACATGAGAAGAGGCTGACGTCTTCAGCCCACCAGTCAGCGCCGAGGAGGCCAGGAGTGGGCCGGCGTGGAGTATTAGGGGCTGCGTAGTAGGTCCCACGGTTGCTGCAGCGAGCCCAGGCGCGCGCGGTGGGGAGTGAGGCTAAATTTCCAATTTATGCACAAGTTTAATCTCTAAATTTCCAATTTATGCACAAGTAAATCTCCATCATGGGATGCAAAGTTCATTTTATTAATTTATCTTGACCCTCTAAAGGTAATATTCAAGCAAGGAATATTCTCTCTATTACTTATTTTCCGAGTTTAAATTTTTACGTATTAGATTTAAAAGAAGATCTAGTCCCGACTTTGAGTCATTTATAGTTAATATTCATGTAAATGCTTATAGTGCATCATTTAACAATATGCTTAATCATTTAGTTGAATAGAAAAATTTTCTATTTCTTCTTTTCTAAAAATGGTTCTTAGTTTTCCAAAATAGTTCTTGGCTTTCAAAATTCCGTCCTCAGTTTTCAATACTCAAGTATAATTTGAGATATCTGAATTTACTATTTTATCTCTTCATATATTTATTTTATTATCATTAATAATTTTTTTCTTACACAGATTTTGGGCCTTACAGCACCTCCATTTCTCACATGTTTTTTCTTAAGTAGAAGATCTCTGTTAGGGGGAGTCTTTTTTTGTCTCTAATGGGGGAGAAAGTTCTTTCTAAAGGATAACACTCATTTAGGGGAAGTAACAATTTGGTGCTTCAATTGGTATTAATTAACAAATCCTTTATGAGGGCAAGTCTTATTTGCTTCCTACATACTTTTAATGTCTTCCTTTTTGGTGGTTGATGCCAAAGGGGGAGAAGTTTAGGGACCAAAGAAAT

>dhAT-Zm24 chr6 dna chromosome:AGPv2:6:13718781:13717571

TATATATATATATATATATATATATATATATATATATATATATATTGTATTTGTTTATAATATCAAGCTACTCGAGTGCTAGTTCAGAATTATTACAATAAGTATGATACATATGAATGATGCATTTTTTTCATGTCCAAAGAAAATATTTATAACCGGCGTTATCTAAGTTTCATGTGCAATAGCGTAAGTTACATATAAAATCACTCTAAGTATCCAAGTTTCAACATAACCAATATTTCACTTTATCATTTTCATGTGGGAACCTGAGGTTATCTTCTTATAAAATGTTTATTAGTCTTGGTAAATCATTCTCAAAAAATGTTTGAACTCAAGGAGTGGCTAAATGATTATGACACTCGTGAAGTCGTGATATGTTTGGAATGGTGTCTTACACGGTATCCGATAAAAAATTCGTTACTGACGCTATCCGTGTCCGACTAGTTACGTATTCGACACGTGGCTATCCGTATTTGCATCCGAGAACATCCGTATTTGTATTCGTATCCGAAGGTATCCGTATTCGAATTCGAATCTGAATAAAAATATGAAAACAAATATGGTTTCTGTGATATCCGTCCGTATTCGATCCGATTACACCCCTAGTAAATCACTCTAAGTATCCAAGTTTCAACATAACTAATATTTCACTTTATCATTTTCATGTGGGAACCTGAGGTTATCTTCTTATAAAATGTTTATTAGTCTTGGTAAATCATTCTCAAAAAATGTTTGGACTCAAGGAGTGCTAAATGATTATGACACTCGTGAAGTCGTGATATGTTTGGAATGGTGTCTTACACGGTATCCGACAAAAAATTCGTTACTGACGCTATCCGTGTCCGACTAGTTCCGTATTCGACACGTGGCTATCCGTATTTGCATCCGAGAACATCTGTATTTGTATTCGTATCCGAAAGTATCTATATTCGAATTCGAATCCGAATAAAAATATGAAAACAAATATGGTTTCTGTGATATCCGTCTGTATTCGATCCGATTACATCCCTGCTTCTTGCAGCGCTCGTCGGCGGCCAGCCAGCACGTGGCCGAACACCTCCGACCTCGCGGCCTGCGTGCATGCGTCAGATCAGACGTGAGATTAATTAGCTGATGGCCTGCCTCGGCTTCGTTTGAGAAGGCCCACGTTGTATGCGTAGAAGGAAAAGCCCAGTAACTTACCAGAATGGCTTTGTGGGCCTATATTGGGG
